# Dynamic Membrane Formation in Anaerobic Dynamic Membrane Bioreactors: Role of Extracellular Polymeric Substances

**DOI:** 10.1371/journal.pone.0139703

**Published:** 2015-10-05

**Authors:** Hongguang Yu, Zhiwei Wang, Zhichao Wu, Chaowei Zhu

**Affiliations:** 1 State Key Laboratory of Pollution Control and Resource Reuse, School of Environmental Science and Engineering, Tongji University, Shanghai 200092, PR China; 2 Chinese Research Academy of Environmental Sciences, Beijing 100012, P.R. China; Duke University Marine Laboratory, UNITED STATES

## Abstract

Dynamic membrane (DM) formation in dynamic membrane bioreactors plays an important role in achieving efficient solid-liquid separation. In order to study the contribution of extracellular polymeric substances (EPS) to DM formation in anaerobic dynamic membrane bioreactor (AnDMBR) processes, EPS extraction from and re-addition to bulk sludge were carried out in short-term filtration tests. DM formation behaviors could be well simulated by cake filtration model, and sludge with EPS re-addition showed the highest resistance coefficient, followed by sludge after EPS extraction. The DM layers exhibited a higher resistance and a lower porosity for the sludge sample after EPS extraction and for the sludge with EPS re-addition. Particle size of sludge flocs decreased after EPS extraction, and changed little with EPS re-addition, which was confirmed by interaction energy analysis. Further investigations by confocal laser scanning microscopy (CLSM) analysis and batch tests suggested that the removal of *in-situ* EPS stimulated release of soluble EPS, and re-added EPS were present as soluble EPS rather than bound EPS, which thus improved the formation of DM. The present work revealed the role of EPS in anaerobic DM formation, and could facilitate the operation of AnDMBR processes.

## Introduction

Anaerobic treatment processes have been widely used for their unique ability to produce energy, generate good soil conditioners and destroy troublesome hazardous chemicals [1]. With the aim for sustainable development in the future, anaerobic processes are expected to play a dominant role in wastewater treatment and sludge digestion. When anaerobic processes are combined with membrane technologies, solid-liquid separation efficiency will be improved. In recent years, dynamic membrane (DM) technology has been adopted as an alternative to traditional microfiltration/ultrafiltration (MF/UF) membranes in membrane bioreactor (MBR) processes as DM has several advantages such as lower membrane costs, lower fouling rate and higher filtration fluxes [2]. DMs, which are also called as secondary membranes, can be formed and reformed in situ by large particles existing in wastewater or mixed liquor on underlying support materials such as meshes or cloth [3]. Anaerobic dynamic membrane bioreactors (AnDMBRs), which couple DM and anaerobic membrane bioreactors, are promising biological treatment processes, and have been applied for treating wastewater, landfill leachate and waste activated sludge [4–6].

In the operation of AnDMBR processes, filtration can be chronologically divided into two stages [4]. At the initial stage of filtration, the filter itself can not reject fine particles in mixed liquor due to the relatively large pores, resulting in the presence of particles in effluent. At the second stage, once DM layer is formed, high-quality effluent, comparable to MF or UF, can be achieved [2, 4, 7–10]. During the filtration process, the initial stage is inevitable, which leads to low-quality effluent when the filtration starts. Thus, studies are needed to overcome the limitation of DM technology. The DM layer can serve as a barrier that limits the passage of fine particles through the support layer. DM formation is the most predominant factor for achieving enhanced performance in AnDMBR processes [2]. The initial stage can be shortened with better DM formation, resulting in high-quality effluent after a short period. For this reason, it is necessary to improve DM formation.

For self-forming dynamic membranes, DM layers are formed by suspended solid particles (such as sludge flocs) present in the bulk solution [2, 4, 7]. Sludge flocs have a loose structure, in which microorganisms are glued together by a three-dimensional matrix of extracellular polymeric substances (EPS). EPS are complex polymers with high molecular weight, which are composed of various organic matters such as polysaccharides, proteins and humic substances. They have special functions in microbial metabolisms. Yu et al. [11] reported that EPS acted as a buffer layer for organic matter transformation through microbial cells. Liu et al. [12] found that EPS mediated cell-cell interactions, facilitated adherence of cell to surface, and were involved in sludge aggregation. EPS also play an important role in membrane fouling. In traditional MBRs, bound EPS were observed to have negative effects on sludge filterability, and demonstrate positive correlations with membrane fouling [13, 14]. Soluble EPS were also proven to induce membrane fouling [15, 16]. In both aerobic and anaerobic MBRs with DMs, EPS were similarly found to have significant impacts on membrane fouling [17, 18]. In DM processes, however, relatively high membrane fouling potential at initial stage can benefit DM formation. Moreover, EPS have been detected in DM layer [4, 7, 17, 19]. Thus, it is essential to investigate the role of EPS in DM formation.

To date, a number of studies have been carried out on DM formation [4, 18, 20]. However, information on the relationship of EPS and DM formation is still limited, especially in an anaerobic environment. Therefore, the overarching goal of this study is to elucidate the role of EPS in DM formation in AnDMBR processes. In this work, we performed the experiments using the anaerobic bulk sludge with *in-situ* EPS (before EPS extraction), without EPS (after EPS extraction) and with EPS (with EPS re-addition). Short-term filtration tests of various sludge samples were conducted in dead-end cells. DM properties were characterized by specific resistance, porosity, particle size and fractal dimension. To further explore the underlying mechanisms, interaction energy and confocal laser scanning microscopy (CLSM) analysis were carried out.

## Materials and Methods

### Bulk sludge fractionation

Sludge samples were taken from the membrane zone of an AnDMBR as described elsewhere [5]. Total suspended solids (TSS) and volatile suspended solids (VSS) concentrations of raw sludge were 19.8±0.3 g/L and 13.6±0.1 g/L, respectively. Analyses of TSS and VSS were conducted based on standard methods [21]. For organic matter determination, polysaccharides were determined by the anthrone method with glucose as a standard reference [22], while proteins and humic substances were measured using the modified Lowry methods using bovine serum albumin and humic acid as standard references, respectively [23]. Extraction of organic matter and EPS was conducted according to our previous publication [11], and the detailed procedure could be found in S1 Text and S1 Fig. Soluble EPS contained 8.1±0.2 mg/L of polysaccharides, 17.2±0.2 mg/L of proteins, and 49.1±0.7 mg/L of humic substances, while polysaccharides, protein and humic substance concentrations of bound EPS were 81.5±3.0, 382.4±15.3 and 439.0±48.6 mg/L, respectively. After sludge fractionation, four sludge samples with the same TSS concentration were obtained, which were AS0 (raw sludge), AS1 (sludge after supernatant decantation but before bound EPS extraction), AS2 (sludge after bound EPS extraction), and AS3 (AS2 with EPS re-addition), respectively.

### Short-term filtration tests

In order to characterize DM formation behaviors, short-term filtration tests were conducted in dead-end filtration mode at constant trans-membrane pressure (TMP). Nylon mesh with an average pore size of 39 μm was chosen for the experiment. Contact angle of the virgin mesh was 113.9±0.8°, implying that it was hydrophobic. For each test, 150 mL of sludge samples was put into a dead-end cell (MSC300, Mosu Corp., China) operated under mesophilic conditions (35±1°C). By applying compressed nitrogen gas, TMP was set at 10, 20 and 30 kPa, respectively. The filtration duration was 90 min. Filtrate was collected in a sampling bottle placed on an electronic balance (YP1002N, Shanghai Precision & Scientific Instrument Co., Ltd., China) and used to calculate permeate flux. Each filtration test was conducted in triplicate, and statistical methods including *t*-test and one-way analysis of variance (ANOVA) were applied for processing TMP and physicochemical data of the DMs.

### Batch tests for EPS transformation analysis

In order to evaluate EPS transformation and existing status in sludge samples, batch tests were conducted as follows. 100 ml of AS1, AS2 and AS3 samples were put into glass assay bottles sealed with rubber stoppers and aluminum foil. Bottles were then flushed with N_2_ gas for 5 min and kept in a shaker (100 rpm) at 35 ± 1°C for 3 h. A pre-determined volume of sludge samples were collected every hour for EPS determination to verify the transformation of *in-situ* EPS and EPS in sludge after EPS extraction and the existing status of re-added EPS.

### Analytical methods

#### Fouling resistance, modeling, DM layer specific resistance and porosity

Fouling resistance can be expressed by Eq (1) according to Darcy’s law.

$$R_{\text{t}}=R_{\text{m}}+R_{\text{f}}=R_{\text{m}}+R_{\text{p}}+R_{\text{c}}=\frac{\Delta P}{\mu J}$$ (1)

In this equation, *R*_t_ is the total resistance (m^−1^), *R*_m_ is the intrinsic resistance of Nylon mesh (m^−1^), *R*_f_ is the fouling resistance (m^−1^), *R*_p_ is the pore-clogging resistance (m^−1^), *R*_c_ is the DM layer resistance (m^−1^), Δ*P* is the trans-membrane pressure (Pa), *μ* is the dynamic viscosity (Pa·s), and *J* is the instantaneous flux (m^3^/(m^2^·s)). *R*_t_ was calculated using the mesh flux at each TMP in each filtration. *R*_m_ was determined by measuring the de-ionized (DI) water flux. *R*_f_ was then obtained by subtracting *R*_m_ from *R*_t_. At the end of the filtration, the mesh surface was physically cleaned with sponge and DI water to remove the cake layer. Subsequently, DI water flux was measured again to evaluate *R*_m_+*R*_p_. In this way, *R*_p_ and *R*_c_ was worked out by using Eq (1).

According to the model proposed by Hermia [24], as shown in Eq (2), four fouling scenarios are responsible for flux decline under constant filtration pressure.

$$\frac{d^{2}t}{dV^{2}}=k{\left(\frac{dt}{dV}\right)}^{i}$$ (2)

where *t* is the filtration time (s), *V* is the filtration volume (m^3^), *i* is the blocking index, and *k* is the resistance coefficient (s^1-i^/m^2-i^). Four fouling scenarios can be described by these parameters as follows: pore constriction *i* = 1.5, intermediate blocking *i* = 1, complete blocking *i* = 2, and cake filtration *i* = 0.

Permeate flux can be expressed by Eq (3).

$$J=\frac{1}{A_{\text{m}}}\cdot \frac{dV}{dt}$$ (3)

where *A*_m_ is the mesh area (m^2^). In this study, the mesh area is 5.02×10^−3^ m^2^.

For cake filtration model (*i* = 0), the change of flux can be described by Eq (4) via using Eqs (2) and (3).

$$\frac{dJ}{dt}=-kA_{\text{m}}^{2}J^{3}$$ (4)

Using the differential equation (Eq (5)) and resistance equation (Eq (1)), resistance can be calculated by Eq (6).

$$dJ=-\frac{\Delta P}{\mu R^{2}}dR$$ (5)

$$R=\frac{A_{\text{m}}\cdot \Delta P}{\mu }\sqrt{2kt}$$ (6)

In this way, cake filtration model can be fitted to obtain the resistance coefficient (*k)*.

The relationship between DM layer resistance (*R*_c_) and specific resistance (*α*) can be expressed by Eq (7) [25].

$$R_{\text{c}}=\frac{\alpha M}{A_{\text{m}}}$$ (7)

where *α* is the DM layer specific resistance (m/kg), and *M* is the mass of DM layer (kg).

If we assume that the particles are rigid spherical, the correlation between DM specific resistance and DM layer porosity can be described by Carman-Kozeny equation [26].

$$\alpha =\frac{180(1-\varepsilon_{\text{p}})}{\rho_{\text{p}}d_{\text{p}}^{2}\varepsilon_{\text{p}}^{3}}$$ (8)

where *ε*_p_ is the porosity of DM layer, *ρ*_p_ is the density of particle (kg/m^3^), and *d*_p_ is the particle diameter (m).

#### Particle size and fractal dimension

At the end of filtration tests, the cake layer (i.e., DM layer) of each test was gently scraped off the mesh surface and collected. Sludge and DM layer samples were immediately analyzed for particle size and fractal dimension using a particle size analyzer (Mastersizer 3000, Malvern Instruments Ltd., UK). Information of fractal dimension measurement has been described elsewhere [27].

#### Interaction energy analysis

For zeta potential determination, sludge was diluted by DI water according to literature [28], and then measured using a Zetasizer analyzer (Nano-ZS90, Malvern Instruments Ltd., UK). By using a contact angle analyzer (JC2000 D1, Powereach Co., China), contact angle of each sample was obtained by averaging at least seven independent measurements. Total interaction energy between sludge flocs was calculated according to the Derjaguin–Landau–Verwey–Overbeek (DLVO) theory using contact angle and zeta potential values [12, 29].

#### Confocal laser scanning microscope (CLSM)

A confocal laser scanning microscope (CLSM) (Nikon A1, Tokyo, Japan) was used to characterize EPS bound to sludge flocs. The components of bound EPS including proteins, α-polysaccharides and β-polysaccharides were stained according to the methods by Chen et al. [30]. After scans with a 60× objective, CLSM images were analyzed with image analysis system (Image J, V1.45s, NIH, USA).

## Results and Discussion

### DM formation behaviors

**Fig 1:**
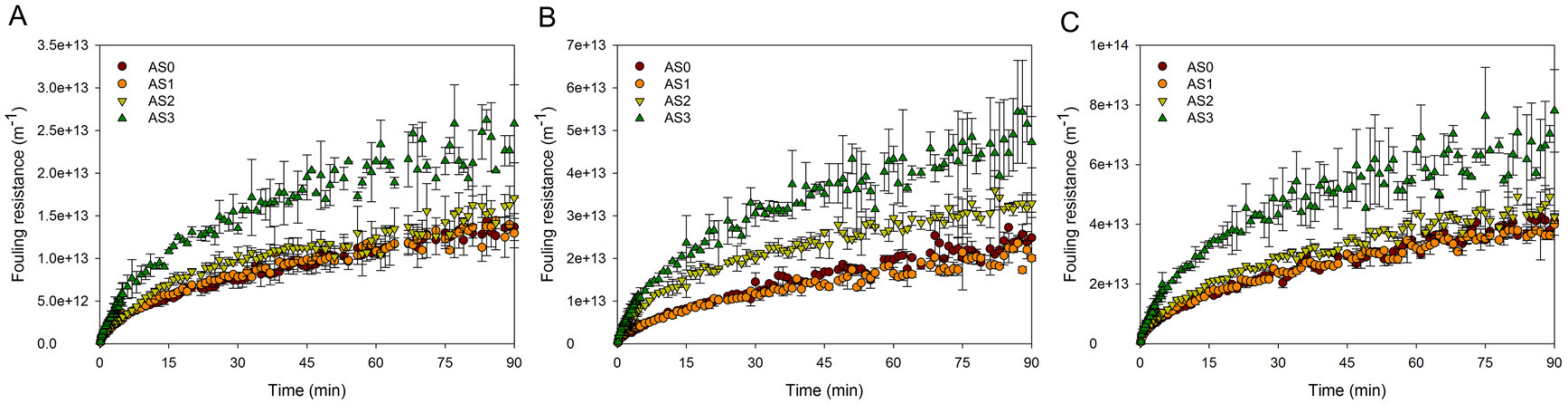
Fouling resistance with time at different TMPs. (A) 10 kPa, (B) 20 kPa, (C) 30 kPa. Error bars represent standard deviations of triplicate tests.

DM forming behaviors were investigated in short-term filtration tests, and fouling resistances of sludge samples at various TMPs are shown in Fig 1. The evolution of fouling resistance was well fitted by Eq (6) (Table 1). All the R^2^ values in the fitting results of cake filtration model were higher than 0.90, and regression analysis was significant at 0.05 level, demonstrating that all the DM formation behaviors in our study can be simulated by cake filtration model on the mesh surface. At each TMP, fouling resistances of sludge samples increased with time (Fig 1), indicating that cake layers kept growing during the filtration. Hwang et al. [31] observed that particle fouling behaviours in dead-end filtration using MF membrane changed with filtration time. In their case, particle fouling was caused by pore blocking at the first stage and cake formation at the second stage. The DM formation mechanisms in our study were different from microfiltration membrane fouling mechanisms. Based on one-way ANOVA, fouling resistances of AS0 (raw sludge) and AS1 exhibited no notable differences at each TMP (*p* = 0.832, 0.215 and 0.807 at 10, 20 and 30 kPa, respectively), indicating sludge after supernatant decantation had no obvious distinctions in DM formation. However, for the rest sludge samples, fouling resistances under the same TMP and filtration time, followed the order of AS3 > AS2 > AS1, suggesting that sludge after bound EPS extraction had a higher fouling rate. After EPS re-addition, the fouling rate appeared even higher. Meanwhile, similar results were observed in terms of resistance coefficient (*k*) values (Table 1). The differences of *k* values between AS0 and AS1 are also not significant according to *t*-test (*p* = 0.736), and *k* values of different sludge samples (Table 1) follow the order of AS3 > AS2 > AS1. In DM formation, higher fouling rate and resistance coefficient led to higher DM formation efficiency. Fouling potential of sludge after EPS extraction was higher than that before EPS extraction, and it became even larger with re-addition of EPS, indicating that re-added EPS were favorable for DM formation. In the following sections, comparisons of AS1, AS2 and AS3 will be analyzed in detail to investigate the role of EPS in DM formation.

**Table 1:** Cake filtration model fitting results^a^.

TMP (kPa)	Resistance coefficient *k* (×10^12^ s/m^2^)			
	AS0*	AS1*	AS2*	AS3*
10	3.52±0.52	3.59±0.10	4.77±0.19	11.85±0.37
20	2.54±0.08	2.01±0.12	5.58±0.27	11.84±0.50
30	3.50±0.07	3.31±0.03	4.76±0.22	11.03±0.51

* Regression analysis was significant (*p*<0.05).

^a^ Values are given as average ± standard deviation (*n* = 3).

### DM layer characteristics

**Fig 2:**
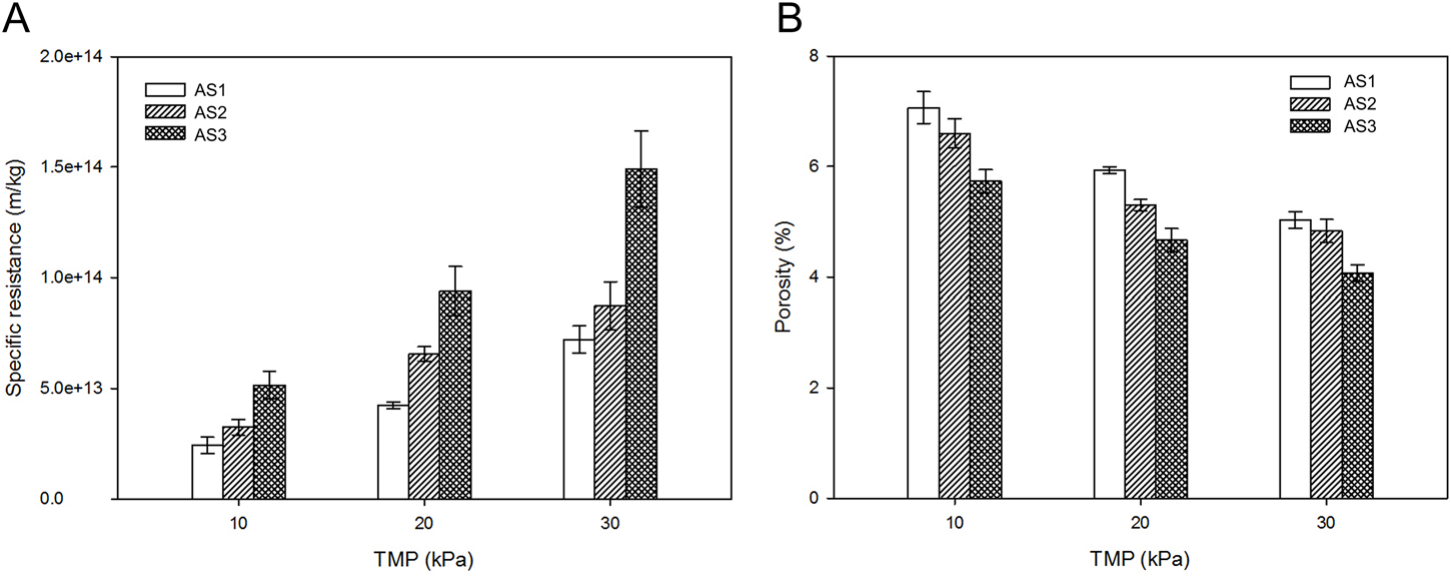
Variations in (A) specific resistance and (B) porosity of DM layer at the end of filtration. Error bars represent standard deviations of triplicate tests.

At the end of the filtration experiments, DM layer (cake layer) specific resistances and porosities of sludge samples were calculated (Fig 2). Specific resistances and porosities were within the range of 2.4×10^13^~1.5×10^14^ m/kg and 4.1~7.1%, respectively. It has been reported that cake layer specific resistance and porosity in traditional MBRs are about 9.5×10^13^ m/kg and 69.6%, respectively [32]. In our study, specific resistances were comparable to those in MBR processes, while the porosities were much smaller, indicating that more compact DM layers were formed in AnDMBR systems. As shown in Fig 2A, specific resistance of each sludge sample increases with TMP, and at the same TMP, DM layer specific resistances follow the order of AS3 > AS2 > AS1. On the contrary, porosities decreases with TMP, and at the same TMP, DM layer porosities are as follows: AS3 < AS2 < AS1. Specific resistances are negatively related to porosities (correlation coefficient was -0.935). In less porous DM layers, it is more difficult for large particles present in mixed liquor to pass through DM layers. DM layers with higher specific resistances and lower porosities are thus able to reject more particles, and better solid-liquid separation could be achieved. The results indicated that DM layers became more suitable for separation after bound EPS extraction, and even better with EPS re-addition.

**Fig 3:**
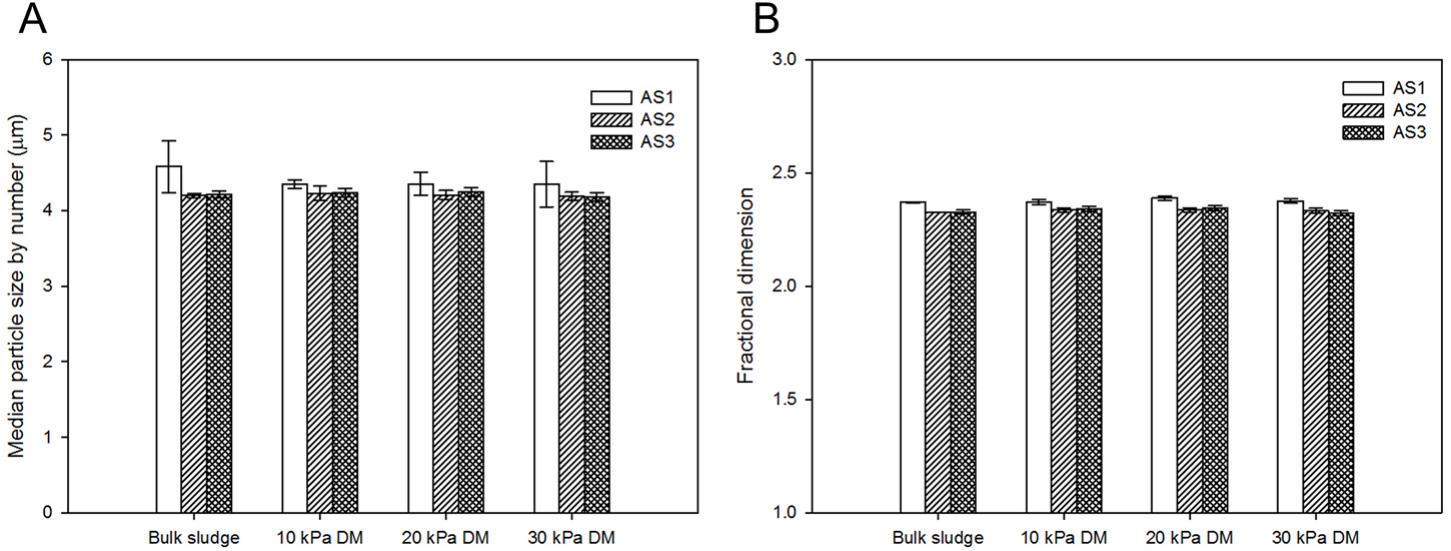
Particle size distribution and fractal dimension of DM layers at various TMPs. (A) median particle size by number, (B) fractal dimension. Error bars represent standard deviations of triplicate tests.

Particle size distribution and fractal dimension of DM layers at different TMPs were further evaluated to understand DM properties (Fig 3). Neither median particle sizes by number nor fractal dimensions of DM layers seemed distinct at different TMPs, indicating that TMP had no significant impacts on particle sizes and structures of DM layers. As seen in Fig 3A, in the case of AS1, AS2 and AS3, no obvious differences of median particle sizes were found between bulk sludge and DM layer (*p* = 0.59, 0.88 and 0.58 in one-way ANOVA of AS1, AS2 and AS3, respectively), which implied that particle sizes hardly changed during the filtration. Meanwhile, in bulk sludge and DM layers, AS2 and AS3 exhibited similar median particle sizes (*p* = 0.437 in t-test), while AS1 contained slightly larger particle sizes (*p* = 0.004 against AS2 and AS3 samples). Yu et al. [33] studied the breakage and regrowth of aerobic sludge flocs, and found that particle sizes decreased after EPS removal and restored to some degree with EPS re-addition. In our study, no restoration of particle size was observed with EPS re-addition, indicating that the properties of anaerobic sludge were distinct from those of aerobic sludge. As shown in Fig 3B, fractal dimensions of bulk sludge and DM layers seemed to remain stable within the range of 2.3–2.4. Fractal dimension has been found to have values in the 2.3–2.5 range for compact aggregates [34]. Therefore, DM layers had condensed structures, which were also supported by specific resistance and porosity results (Fig 2). It was also found that fractal dimensions of sludge had almost no change during DM formations at various TMPs, indicating that interspace between sludge flocs might be condensed to form DM layers, but the flocs themselves were not restructured during DM formation.

### Role of EPS in DM formation

In order to further clarify the role of EPS in anaerobic DM formation, physicochemical properties of sludge flocs were investigated. Zeta potential values of AS1, AS2 and AS3 were -29.5±1.3, -31.3±1.2 and -32.8±1.1 mV (*n* = 3), respectively. No significant difference in zeta potential between AS2 and AS3 was observed (*p* = 0.174 in *t*-test), and the absolute zeta potential value of AS2 was slightly larger than that of AS1. Results showed that sludge flocs became more difficult to aggregate after bound EPS extraction (*p* = 0.042 in one-way ANOVA), which might result in smaller particle sizes of AS2. However, re-addition of EPS seemed to have no effects on sludge aggregation, which led to no change in particle sizes (Fig 3A).

**Fig 4:**
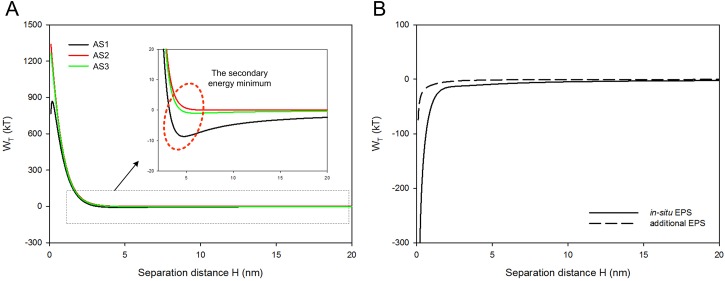
Total interaction energy curves of (A) sludge and (B) EPS.

The interaction energies between sludge cells were calculated according to DLVO theory. As shown in Fig 4A, total interaction energy curves between AS2 and AS3 were not notably distinct (*p* = 0.776 in one-way ANOVA test), indicating that re-addition of EPS had little impact on the interactions between sludge flocs. Meanwhile, energy barriers (maximum energies) of three sludge samples were more than 800 kT. The energy barrier increased by 54% after bound EPS extraction, while scarcely changed with EPS re-addition. The dispersed sludge flocs needed sufficient energy to overcome the energy barrier for aggregation [12]. Therefore, these results indicated that higher energy barriers led to more stable sludge flocs with smaller particle sizes (Fig 3A).

The secondary energy minimum in the interaction energy profiles represent the desorption ability of sludge cells from sludge surfaces [35]. As the potential well of the secondary energy minimum value improved, the energy to disperse the sludge cells increased, thus enhancing the stability of sludge structure [12]. From Fig 4A, it can be observed that both the secondary energy minimum values of AS2 and AS3 were higher than that of AS1, again supporting the hypothesis that AS2 and AS3 had enhanced sludge floc stability. During DM formation, sludge flocs with strong stability were difficult to break down or restructure, and invulnerable to TMPs (Fig 3). Based on total interaction energy curves of sludge, contributions of *in-situ* EPS and re-added EPS were calculated by subtracting total interaction energy of AS2 from that of AS1, and that of AS2 from that of AS3, respectively. As shown in Fig 4B, interaction energy curves of EPS were negative, and interaction energy of re-added EPS was much lower than that of *in-situ* EPS. Results implied that the role of both *in-situ* EPS and re-added EPS was attractive, but re-added EPS had weaker attraction. Sludge structure became more stable and harder for aggregation after bound EPS extraction (*p* = 0.034 in one-way ANOVA for AS1 and AS2 samples), while with EPS re-addition, the sludge structure barely changed. The secondary energy minimum and total interaction energy analysis can well explain the insignificant changes in particle size and fractal dimension of bulk sludge and DM layers (Fig 3).

**Fig 5:**
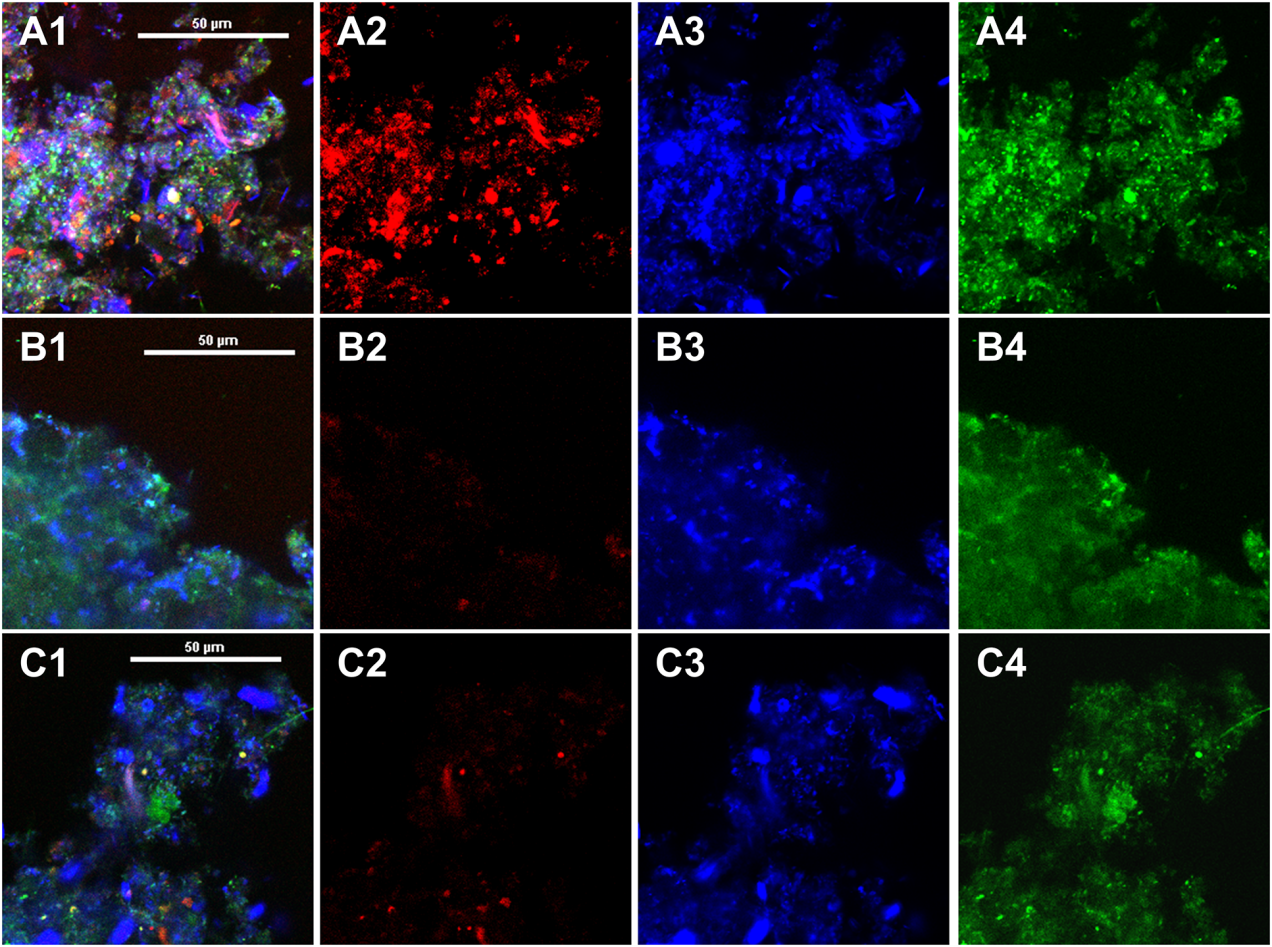
CLSM images of AS1 (A1-A4), AS2 (B1-B4) and AS3 (C1-C4). Symbols 1–4: 1 exhibits combination of individual images in 2–4, 2 represents CLSM image of α-polysaccharides (Con A), 3 represents CLSM image of β-polysaccharides (Calcofluor white), and 4 represents CLSM image of proteins (FITC).

CLSM images of stained sludge flocs were applied to directly visualize the component distributions in EPS bound to flocs (Fig 5). Meanwhile, the relative amount of different components was analyzed via calculating the percentage of specific color in the whole area using Image J, and the results are shown in S2 Fig The relative amount of various components (α-polysaccharides, β-polysaccharides and proteins) decreased after bound EPS extraction, while no increase of the abundance was observed with EPS re-addition, suggesting that the re-added EPS were no longer bound to sludge cells.

**Fig 6:**
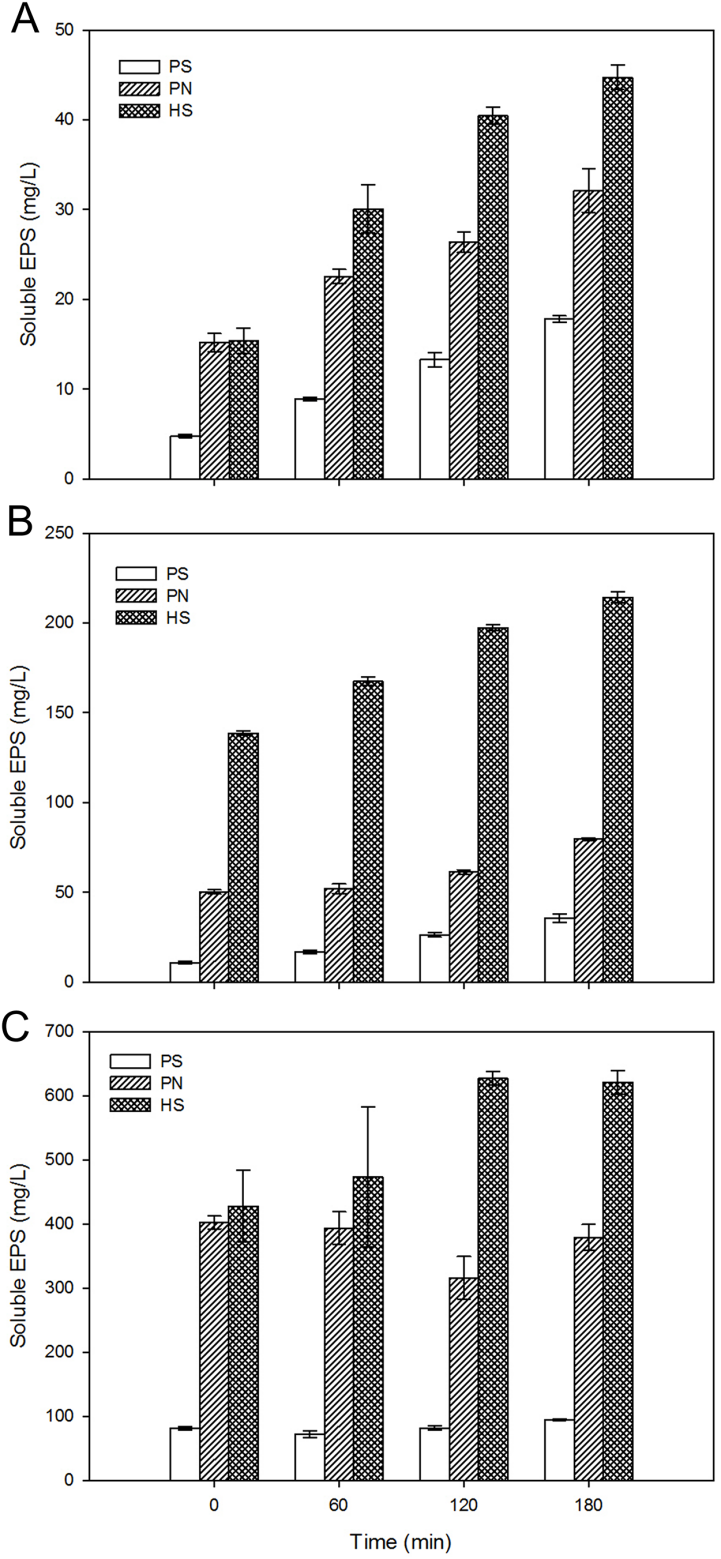
Variations of soluble EPS during short-term batch tests. (A) AS1, (B) AS2, (C) AS3. PS-polysaccharides, PN-proteins, HS-humic substances. Error bars represent standard deviations of triplicate tests.

To further verify EPS existing status of AS3 with re-added EPS, batch experiments were carried out. Meanwhile, AS1 and AS2 samples were also used for comparison. As seen in Fig 6C, the initial soluble EPS concentration of AS3 was similar to the extracted bound EPS concentration, suggesting that re-added EPS existed in soluble status rather than bound status, which is also supported by CLSM images (Fig 5). During the batch test, polysaccharides of AS3 remained relatively stable, while concentrations of proteins and humic substances fluctuated (Fig 6C), unlike the case with AS1 and AS2. The total soluble EPS concentration kept increasing. These results may indicate that soluble EPS of AS3 were released with time and re-added EPS might not be bound to sludge cells. In other words, the occurrences of *in-situ* EPS and re-added EPS were different. *In-situ* EPS appeared as bound state, while re-added EPS were present as soluble state. Results also suggest that the adsorption and desorption of bound EPS was an irreversible process, which might be because that bound EPS were likely linked to sludge cells by chemical bonds [33]. As for AS1 and AS2, soluble polysaccharides, proteins and humic substances increased with time, indicating that soluble EPS were secreted during batch tests. Moreover, soluble EPS of AS2 were higher than those of AS1 at the same time. One possible explanation might be that after EPS extraction, the substrate was in short supply. Bound EPS would be secreted by sludge cells [36], and hydrolyzed to biomass-associated products (part of soluble EPS) for carbon and energy source [37, 38]. In this way, soluble EPS constituents of AS2 were higher than those of AS1. In summary, the contents of soluble polysaccharides, proteins and humic substances at the same time followed the same order of AS3 > AS2 > AS1 (Fig 6). EPS in soluble state have been found responsible for membrane fouling [39], which is also thought to be positively related to DM formation. EPS in soluble status might penetrate into inter-particle voids of DM layers, resulting in the decrease of porosities and the increase of resistances (Figs 1 and 2).

**Fig 7:**
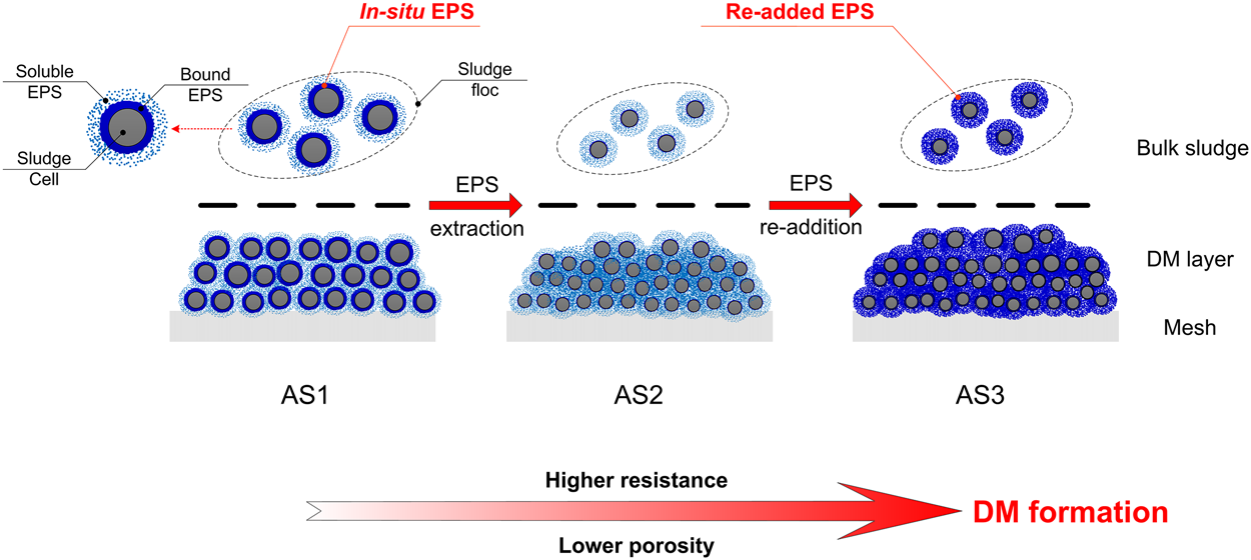
Schematic of anaerobic DM formation with EPS extraction and re-addition.

Based on the obtained results as mentioned above, we proposed a scenario to illustrate the role of EPS in anaerobic DM formation (Fig 7). DM formation behavior was caused by cake formation on the mesh surface. During anaerobic DM formation, interspace between sludge cells in the bulk sludge was condensed to form compact DM layers, in which sludge cells were not restructured. After EPS extraction, sludge cells slightly broke down and became more stable. Meanwhile, removal of *in-situ* EPS stimulated the secretion of more soluble EPS from sludge cells. The released soluble EPS could penetrate into the inter-particle voids of the DM layer, which led to higher resistance and lower porosity. In this way, the DM layer was more compact and DM formation was improved. It was also found that EPS extraction might be irreversible. The re-added EPS were present as soluble EPS rather than bound EPS. Large amounts of soluble EPS resulted in the decrease of DM layer porosity and further facilitated DM formation.

### Implications of this work

EPS are found to affect the properties of sludge and have various functions in microbial metabolism. However, the roles of EPS in DM formation are not clear. In the present work, the contribution of EPS to DM formation of AnDMBR processes was elucidated. Our study implied that in the operation of AnDMBR processes, stimulating EPS release might be feasible to facilitate DM formation at the initial stage of DM formation, e.g., increase of turbulence [39] or ultrasound irritation. On the other hand, based on our obtained results, sludge before bound EPS extraction showed lower fouling resistance than sludge after bound EPS extraction, suggesting that the presence of *in-situ* bound EPS (without extraction) might be beneficial to membrane fouling control for long term operation after DM formation.

## Conclusions

In this study, the roles of EPS in DM formation of AnDMBR processes were evaluated by extraction and re-addition of EPS. After EPS extraction, the DM layer was formed with higher resistance and lower porosity. The DM layer compactness was further increased with EPS re-addition. Particle size of sludge flocs slightly decreased after bound EPS extraction, while it changed little with EPS re-addition, which was confirmed by interaction energy analysis. Further investigation implied that the removal of *in-situ* EPS stimulated soluble EPS release, and re-added EPS were present as soluble state rather than bound state, which might be responsible for the improved DM formation.

## Supporting Information

S1 Fig Sludge fractionation procedure. (TIF)

S2 Fig Stained area percentages of various sludge samples based on CLSM images. (TIF)

S1 Text Bulk sludge fractionation protocol. (DOC)

## Abbreviations

AnDMBR: anaerobic dynamic membrane bioreactor
ANOVA: analysis of variance
AS0: raw anaerobic sludge
AS1: anaerobic sludge before bound EPS extraction
AS2: anaerobic sludge after bound EPS extraction
AS3: AS2 with EPS re-addition
CLSM: confocal laser scanning microscopy
DI: de-ionized
DLVO: Derjaguin–Landau–Verwey–Overbeek
DM: dynamic membrane
EPS: extracellular polymeric substances
MBR: membrane bioreactor
MF: microfiltration
PBS: phosphate-buffered saline
TMP: trans-membrane pressure
TSS: total suspended solids
UF: ultrafiltration
VSS: volatile suspended solids
*A*_m_: mesh area (m^2^)
*d*_p_: particle diameter (m)
*i*: blocking index
*J*: instantaneous flux (m^3^/(m^2^·s))
*k*: resistance coefficient (s^1-i^/m^2-i^)
*M*: mass of DM layer (kg)
*R*_c_: cake layer (DM layer) resistance (m^−1^)
*R*_f_: fouling resistance (m^−1^)
*R*_m_: intrinsic resistance of Nylon mesh (m^−1^)
*R*_p_: pore-clogging resistance (m^−1^)
*R*_t_: total resistance (m^−1^)
*t*: filtration time (s)
*V*: filtration volume (m^3^)
*A*: DM layer specific resistance (m/kg)
Δ*P*: trans-membrane pressure (Pa)
*ε*_p_: DM layer porosity
*μ*: dynamic viscosity (Pa·s)
*ρ*_p_: particle density (kg/m^3^)
