# Supplementary figures and images for: Dynamic Membrane Formation in Anaerobic Dynamic Membrane Bioreactors: Role of Extracellular Polymeric Substances
Source: PLoS One. 2015 Oct 5;10(10):e0139703. doi: 10.1371/journal.pone.0139703 (PMC4593540; doi:10.1371/journal.pone.0139703)

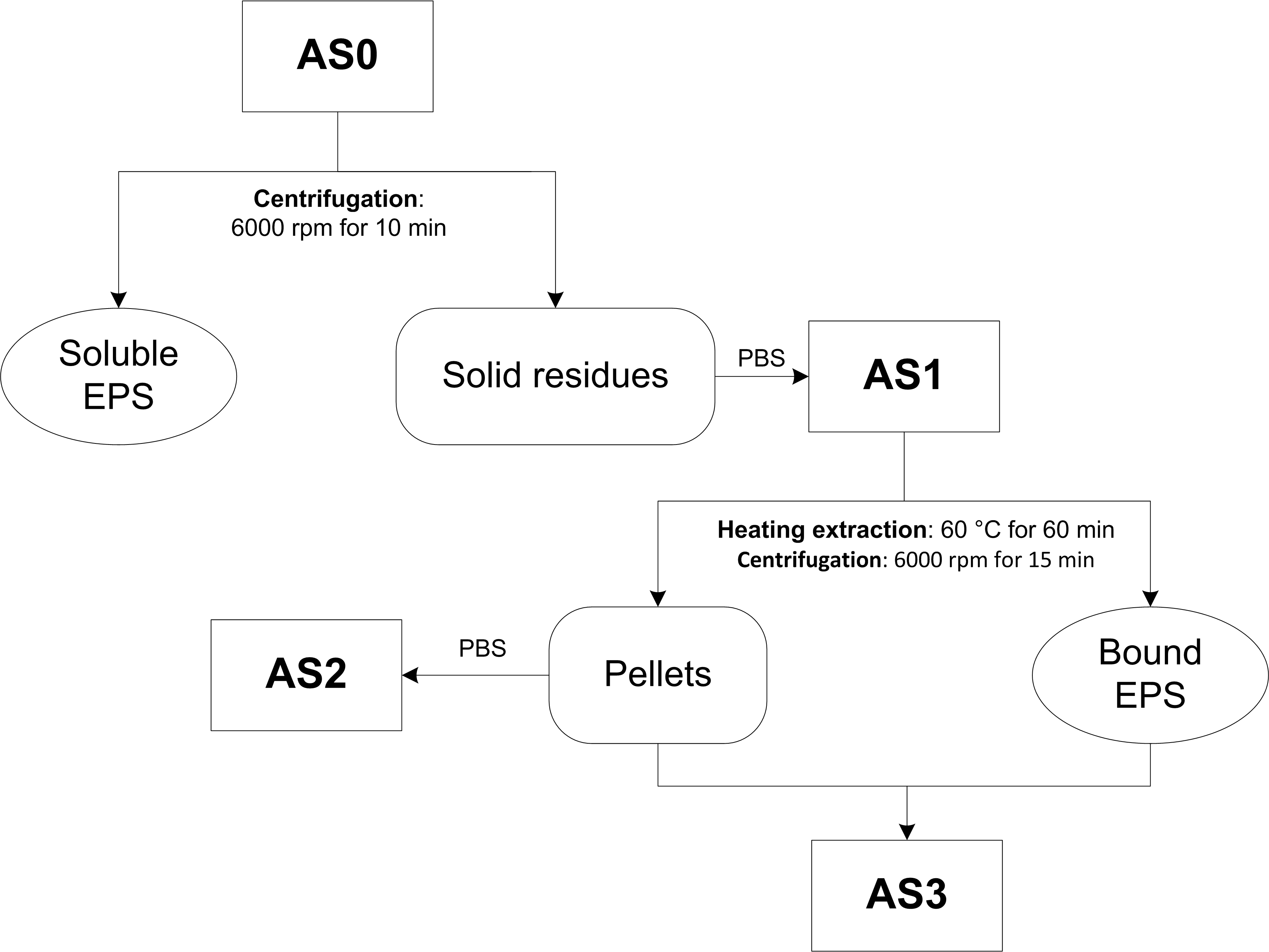

Supplement: S1 Fig — (TIF) [file pone.0139703.s001.tif]

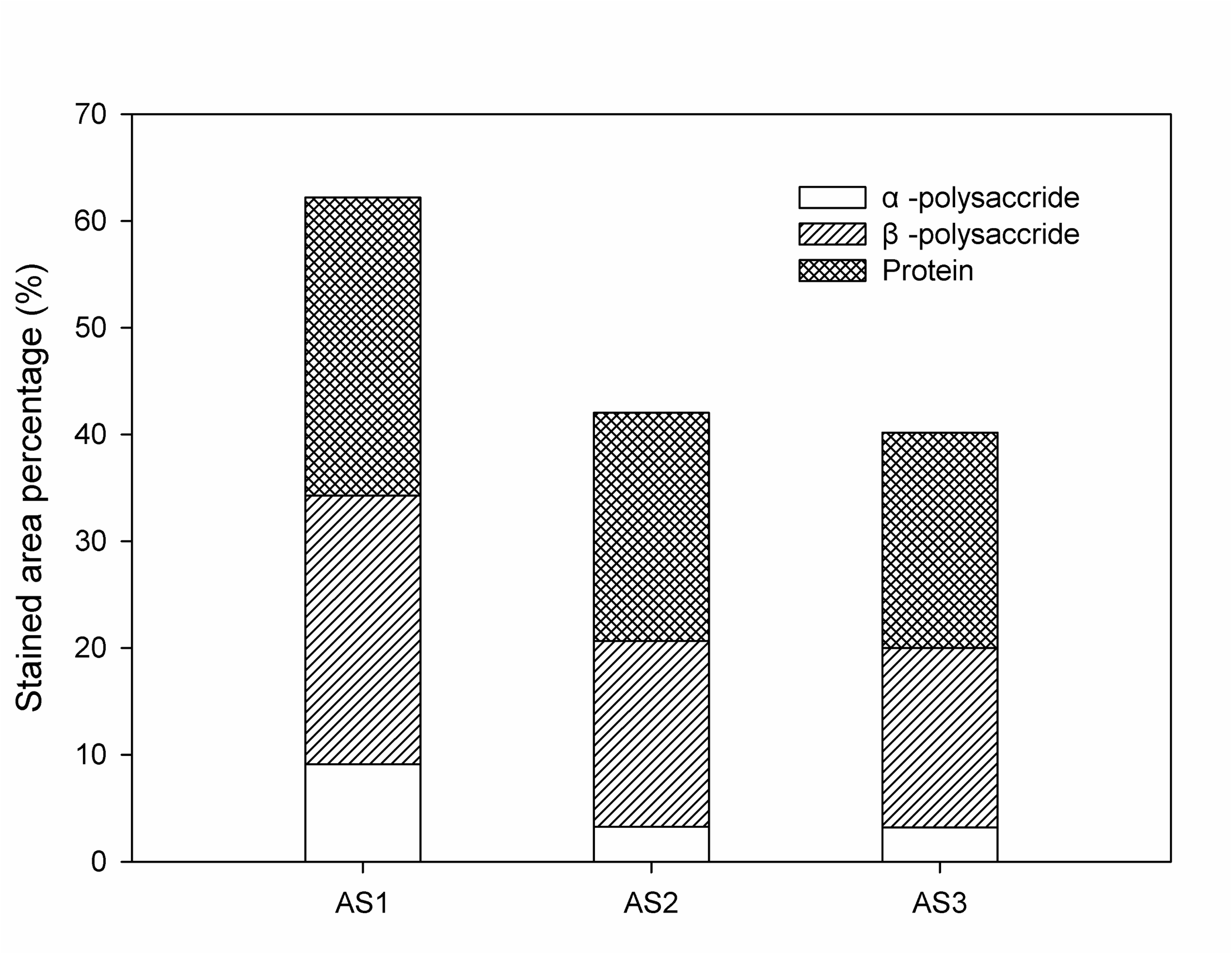

Supplement: S2 Fig — (TIF) [file pone.0139703.s002.tif]
