# Supplementary material for: Dynamic Membrane Formation in Anaerobic Dynamic Membrane Bioreactors: Role of Extracellular Polymeric Substances
Source: PLoS One. 2015 Oct 5;10(10):e0139703. doi: 10.1371/journal.pone.0139703 (PMC4593540; doi:10.1371/journal.pone.0139703)
Supplement: S1 Text — (DOC) [file pone.0139703.s003.doc]

Supporting Information

**Dynamic membrane formation in anaerobic dynamic membrane bioreactors: Role of extracellular polymeric substances**

Hongguang Yu, Zhiwei Wang*, Zhichao Wu

State Key Laboratory of Pollution Control and Resource Reuse, School of Environmental Science and Engineering, Tongji University, Shanghai 200092, PR China

*Corresponding author. Tel/Fax: +86(21)65980400; E-mail address: zwwang@tongji.edu.cn (Z.W. Wang)

This Supporting Information contains three pages and two figures.

1. **Bulk sludge fractionation protocol**

Sludge fractionation was conducted as follows (Figure S1). Raw sludge (AS0) samples were centrifuged at 6000 rpm for 10 min. The supernatant was filtered through normal quantitative papers, and the organic matter in the filtrate represented soluble EPS (supernatant), which contained 8.1±0.2 mg/L of polysaccharides, 17.2±0.2 mg/L of proteins, and 49.1±0.7 mg/L of humic substances. The residue solids were re-suspended to their original volume with phosphate-buffered saline (PBS, pH 7.2), and the mixed liquor was named AS1, i.e., sludge before bound EPS extraction. AS1 was then heated at 60 °C for 60 min, and centrifuged at 6000 rpm for 15 min. The supernatant was filtered with normal quantitative papers and the organic matter in the filtrate was bound EPS, of which polysaccharides, protein and humic substance concentrations were 81.5±3.0, 382.4±15.3 and 439.0±48.6 mg/L, respectively. The collected pellets were re-suspended with PBS solution to the original volumes and called AS2, i.e., sludge after bound EPS extraction. Part of the collected pellets was mixed with bound EPS, and the mixture was named AS3, i.e., sludge after bound EPS extraction with EPS re-addition. TSS concentration of AS1, AS2, AS3 was maintained at the same TSS as AS0.

**Figure S1.** Sludge fractionation procedure.

1. **CLSM analysis**


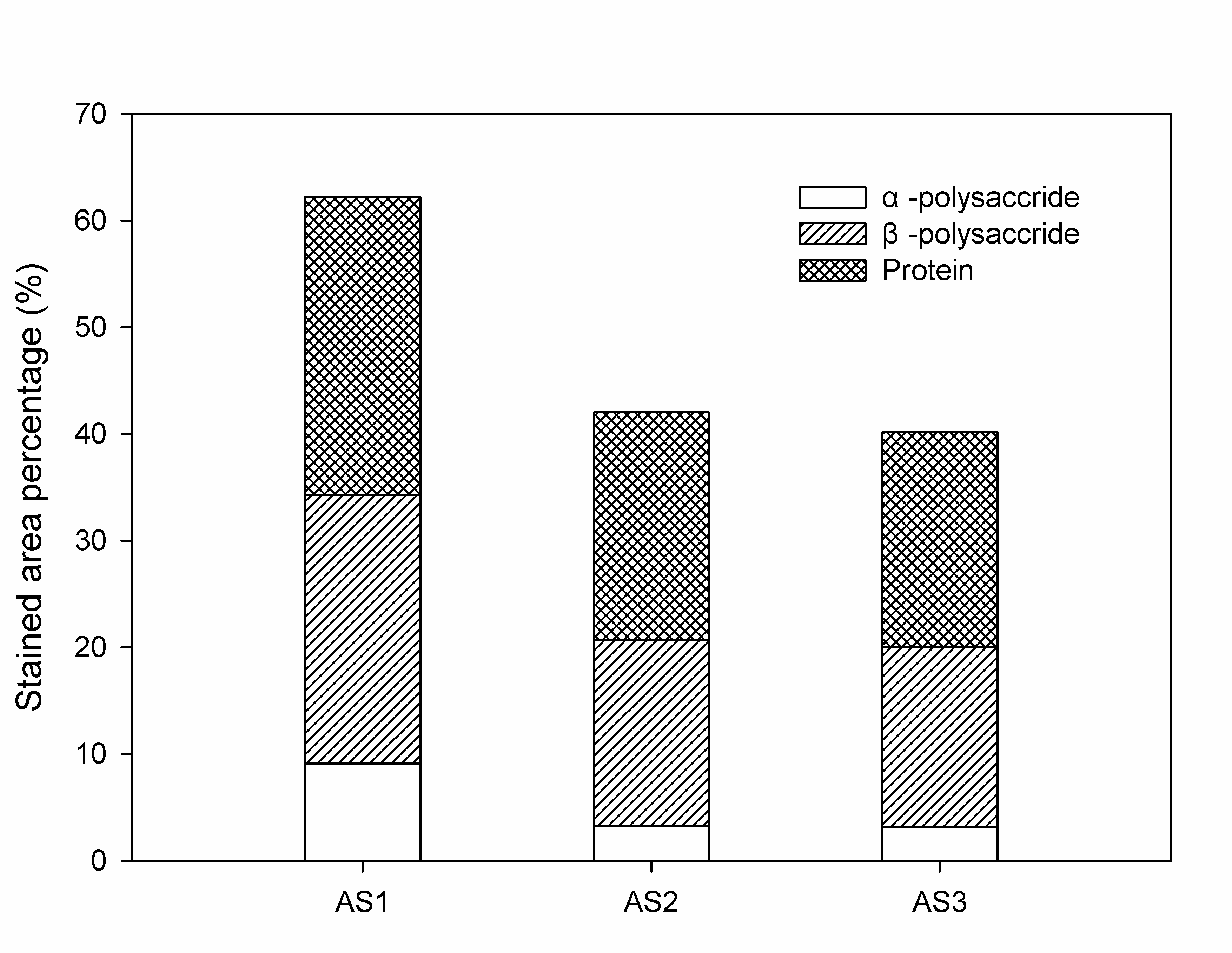


**Figure S2.** Stained area percentages of various sludge samples based on CLSM images.
